# Photodynamic inactivation of *Leishmania braziliensis* doubly sensitized with uroporphyrin and diamino-phthalocyanine activates effector functions of macrophages in vitro

**DOI:** 10.1038/s41598-020-74154-1

**Published:** 2020-10-13

**Authors:** Rohit Sharma, Sayonara M. Viana, Dennis K. P. Ng, Bala K. Kolli, Kwang Poo Chang, Camila I. de Oliveira

**Affiliations:** 1 Instituto Gonçalo Moniz, FIOCRUZ, Salvador, BA Brazil; 2 Chinese University of Hong Kong, Shatin, Hong Kong; 3 Chicago Medical School/Rosalind Franklin University of Medicine & Science, North Chicago, IL 60064 USA; 4 INCT—Instituto de Investigação Em Imunologia, São Paulo, Brazil

**Keywords:** Parasite host response, Parasitic infection

## Abstract

Photodynamic inactivation of *Leishmania* has been shown to render them non-viable, but retain their immunological activities. Installation of dual photodynamic mechanisms ensures complete inactivation of species in the *Leishmania* subgenus, raising the prospect of their safe and effective application as whole-cell vaccines against leishmaniasis. Here, we report the successful extension of this approach to *L. braziliensis* in the *Viannia* subgenus, viz. genetic engineering of promastigotes for cytosolic accumulation of UV-sensitive uroporphyrin (URO) and their loading with red light excitable phthalocyanines (PC) that was cationized by chemical engineering. The transgenic strategy used previously produced *L. braziliensis* transfectants, which gave the same phenotype of aminolevulinate (ALA)-inducible uroporphyria as found in *Leishmania* subgenus, indicative of pre-subgenus evolutionary origin for similar genetic deficiencies in porphyrin/heme biosynthesis. In the present study, 12 independent clones were obtained and were invariably ALA-responsive, albeit to different extent for uroporphyrinogenesis and UV-inactivation. In a separate study, *L. braziliensis* was also found, like other *Leishmania* spp., to take up diamino-PC (PC2) for red light inactivation. In vitro interactions of a highly uroporphyrinogenic clone with primary macrophages were examined with the intervention of URO/PC2-medated double-photodynamic inactivation to ascertain its complete loss of viability. Doubly sensitized *L. braziliensis* transfectants were photo-inactivated before (Strategy #1) or after (Strategy #2) loading of macrophages. In both cases, macrophages were found to take up *L. braziliensis* and degrade them rapidly in contrast to live *Leishmania* infection. The effector functions of macrophages became upregulated following their loading with *L. braziliensis* photodynamically inactivated by both strategies, including CD86 expression, and IL6 and NO production. This was in contrast to the immunosuppressive infection of macrophages with live parasites, marked by IL10 production. The results provide evidence that photodynamically inactivated *L. braziliensis* are susceptible to the degradative pathway of macrophages with upregulation of immunity relevant cytokine and co-stimulatory markers. The relative merits of the two loading strategies with reference to previous experimental vaccination were discussed in light of the present findings with *L. braziliensis*.

## Introduction

Leishmaniases are neglected tropical diseases (NTDs) caused by protozoan pathogens of *Leishmania *spp. Human leishmaniasis has a prevalence of 14 million cases and an estimated 350 million people are at risk of infection worldwide. Nearly 1.3 million new cases and 20,000–30,000 deaths occur annually^1^, the severity being second only to malaria^2^. In Brazil, *Leishmania braziliensis* is the leading cause of cutaneous leishmaniasis (CL), characterized by the development of skin ulcers. Metastasis of *L. braziliensis* to oro-nasopharyngeal cavity causes mucocutaneous leishmaniasis (MCL), a debilitating form of leishmaniasis associated with extensive facial tissue destruction^3^.

Clinical managements of leishmaniasis have relied mainly on chemotherapy with pentavalent antimonials, which are toxic^4^ and ineffective due to emergence of drug-resistance^5^. Thus, the development of an effective vaccine remains a crucial area of research. This has long been considered as feasible, since individuals recovered from leishmaniasis are known to develop life-long immunity. Indeed, numerous efforts have been made and are still on-going to develop such vaccines in different formats, i.e. killed, virulence-attenuated and genetically modified parasites, cDNAs and recombinant peptides plus adjuvants^6^. Vaccination of naïve population with live attenuated parasites is expected to mimic natural infection to elicit protective immunity, but produce no disease. *Leishmania* has been attenuated, for example, by long-term in vitro culture to select for avirulence^7^, and gamma irradiation^8^ and genetic modifications^9–11^ to keep them viable and infective, but non-replicative. Although such attenuated live vaccines have been shown to protect experimental animals against challenges with virulent *Leishmania*, their potential application to human vaccination still awaits safety and efficacy evaluation.

We have been exploring inactivation of *Leishmania* via the principle of photodynamic therapy (PDT) as a strategy to render them non-viable for safe and effective use experimentally not only as whole-cell vaccines against leishmaniasis^12,13^ but also as carriers for vaccines against other infectious and malignant diseases^14–17^. Photodynamic therapy makes use of dyes as photosensitizers (PS) that are excitable by light of specific wavelength to produce biocidal radicals, like singlet oxygen (^1^O_2_) and reactive oxygen species (ROS). *Leishmania* are photodynamically inactivatable to completion when doubly loaded with two different PS, i.e. uroporphyrin (URO) and phthalocyanines (PC). *Leishmania* and other trypanosomes are incapable of porphyrin/heme biosynthesis and acquire heme as an essential nutrient from exogenous sources. Porphyrinogenic *Leishmania,* e.g. *L. amazonensis* was previously produced by complementation of such genetic defects in promastigotes with mammalian cDNAs to express the 2nd and 3rd enzymes in the classic heme biosynthetic pathway, i.e. aminolevulinate dehydratase (ALAD) and porphobilinogen deaminase (PBGD), respectively^18,19^. Exposure of these doubly transfected parasites to aminolevulinate (ALA), the product of the 1st enzyme, results in their cytosolic accumulation of uroporphyrin I (URO)—a PS excitable with longwave UV to produce ^1^O_2_ and other cytotoxic oxidative metabolites secondarily. Vaccination of hamsters with porphyrinogenic *L. amazonensis* followed by ALA treatment and light exposure in vivo conferred protection against the challenge with virulent *L. donovani* and the immunity was adaptively transferrable from immunized hamsters to naïve animals^12^. Sensitization of *L. amazonensis* by a combination of endogenously accumulated URO and exogenously supplied cationic PC provided assurance of their complete photo-inactivation without exception^20^. These *Leishmania* are invariably non-viable, but remain structurally and immunologically intact, as their use to immunize highly susceptible BALB/c mice protected them against a homologous challenge by suppressing lesion development and significantly reducing parasite burdens^13^.

In this study, *L. braziliensis* was successfully transfected to produce uroporphyrinogenic mutants for the first time as a member in the *Viannia* subgenus. Previously, such transfectants have been produced only for members of the *Leishmania* subgenus, i.e. *L. infantum*, *L. major*, *L. tropica* and *L. amazonensis*^18^. A total of 12 *L. braziliensis* transfectant clones were obtained. All were found responsive to ALA for cytosolic URO accumulation, rendering them sensitive to UV-inactivation. The porphyric mutants were further sensitized with a diamino-PC (PC2) for evaluation of their interactions with bone marrow-derived macrophages. The doubly photo-inactivated mutants were effective to elicit oxidative burst of the macrophages as well as to upregulate the production of relevant cytokine and co-stimulatory markers, in support of their potential use as safe and effective vaccines and vaccine carriers.

## Results

### Aminolevulinate (ALA)-induction of the doubly transfected *L. braziliensis* for neogenesis of cytosolic uroporphyrin 1 (URO), indicative of ALAD and PBGD expression

**Figure 1:**
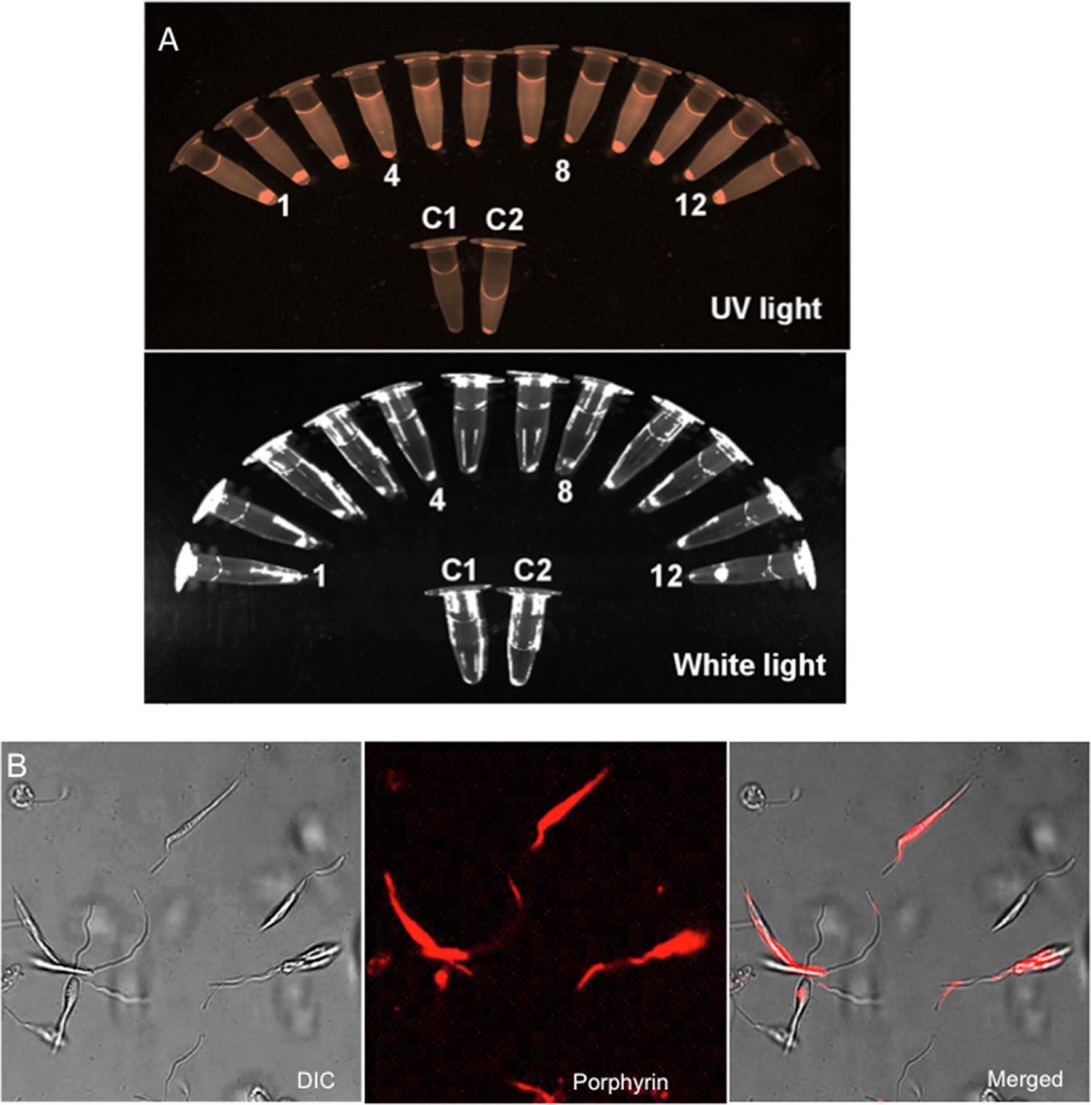
Aminolevulinate (ALA)-mediated porphyrinogenesis of genetically complemented *L. braziliensis* clones. Twelve clones from *L. braziliensis* promastigotes transfected to express PBGD and ALAD were exposed in the dark to ALA (1 mM) for 48 h. (**A**) Cell samples were centrifuged and illuminated with longwave UV (upper panel) or white light (lower panel), showing co-sedimentation of URO fluorescence with cells. Wild type *L. braziliensis* (C1) and porphyric *L. amazonensis* (C2) were similarly processed to serve as negative control and positive control, respectively.(**B**) Microscopic images of porphyric *L. braziliensis*, showing cytoplasmic URO accumulation. DIC, Differential interference; Porphyrin, URO fluorescence; Merged, merging of the two images.

*L. braziliensis* promastigotes were doubly transfected and cloned, as described (see “Methods”). All 12 independent clones obtained grew stably during repeated cycles of passages under the selective drug pressures. They were fully revivable without losing drug-resistance phenotypes after cryopreservation in liquid nitrogen, further indicative of the transgenic stability. Cells of all 12 clones were found responsive to ALA, producing intense porphyrin-specific fluorescence in the cell pellets after centrifugation and illumination with UV lamp (Fig. 1A, top panel 1–12 plus a positive control C2). No fluorescence was visible in those of the wildtype without transfection (Fig. 1A,C1) or when illuminated with white light (Fig. 1A, bottom panel). Notably, all ALA-exposed transfectant clones remained intact and motile, similar to untreated controls when kept in the dark, as noted by phase contrast microscopy of uroporphyric transfectants. Under this setting of very dim illumination, the promastigotes were seen to remain motile initially for ~ 1 s followed by an abrupt cessation of their flagellar motility, in keeping with the lag time expected from excitation of their cytosolic URO to the production of a sufficient amount of cytotoxic ^1^O_2_. Co-sedimentation of the URO fluorescence with cells in pellets after centrifugation is indicative of its cell-association, as noted with similarly treated uroporphyic *L. amazonensis* (Fig. 1A, top panel, clones 1–12 vs. C2). The faint fluorescence observed in the supernatants was presumably due to efflux of URO from uroporphyric transfectants, as seen previsouly^19^. Notably, WT *L. brasiliensis* produced no enzymes equivalent to the trangene products, i.e. ALAD and PBGD, as exposure of these cells to ALA produced no porphyrin fluoresence (Fig. 1A, top panel, C1). Fluorescence microscopy of live transfectants showed that porphyrin was diffused cytosolically throughout the cells, including flagella, but also condensed in cytoplasmic vacuoles (Fig. 1B).

**Figure 2:**
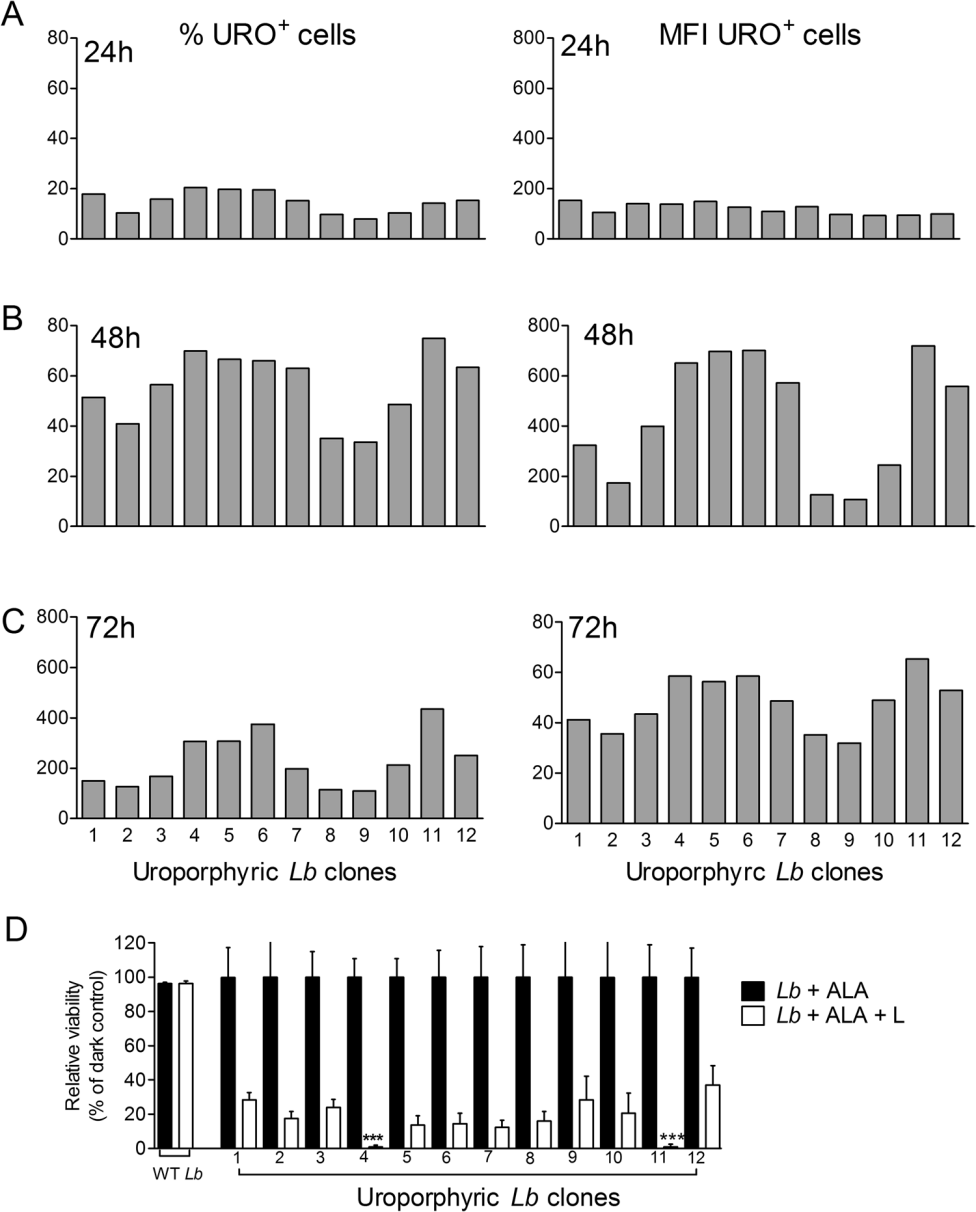
Variation of the 12 transgenic *L. braziliensis* clones during their ALA-induced porphyrinogenesis and susceptibility of all 12 porphyric clones to photo-inactivation. Flow cytometry of 12 doubly transfected *L. braziliensis* clones, showing their variations in the percentage of porphyric cells (left panel) and mean URO-fluorescent intensity (right panel) during ALA-induced porphyrinogenesis for 24 h (**A**), 48 h (**B**) and 72 h (**C**). To determine light-susceptibility (**D**), **c**ells were first incubated with ALA (1 mM) in the dark for 48 h and then split into two sets: one set was kept in the dark (**black bar, Lb + ALA**) and the other set exposed to longwave UV for 30 min (**white bar, Lb + ALA + L**). Cell viability was assessed by MTT reduction assay. Photo-inactivation of UV-exposed cells for each clone was expressed as % of the corresponding dark control. Negative controls: wild-type *L. braziliensis* **(WT Lb)** similarly incubated with ALA followed by exposure (**white bar**) or no exposure (**black bar**) to UV. Data are presented as mean ± S.D. from a representative experiment performed in quadruplicate. ***p < 0.001.

The 12 clones obtained were found to vary in their response to ALA for uroporphyrinogenesis in both kinetics and peak levels, as shown by flow cytometric analysis of these cell samples. Changes were noted in the percentage of porphyric cells and their mean fluorescence intensity (MFI) with the progression of time. URO fluorescence of all ALA-exposed samples began to emerge in ~ 24 h at low levels, i.e. 10–20% porphyric cells and up to ~ 200 MFI in URO-positive cells (Fig. 2A) and peaked in 48 h to variable levels, i.e. 40–70% porphyric cells and 100–700 MFI in URO-positive cells, being lower and the lowest for clones 2–3 and clones 8–9, respectively (Fig. 2B). By 72 h, URO levels decreased in the cells of all 12 clones according to both % porphyric cells and MFI values (Fig. 2C), especially the latter (right panel). This was coincident with the emergence of fluorescence in the supernatants or release of URO, as noted after sedimentation of porphyric cells (Fig. 1A, top panel). Clones # 4–6 and 11–12 were more responsive than clones 2–3 and 8–10 to ALA exposure for porphyrinogenesis.

All 12 porphyric clones were sensitive to inactivation by UV illumination, as shown by the MTT reduction assay for cell viability. Their MTT reduction activities decreased to 20–30% of the respective dark controls in most cases (Fig. 2D, white bar vs. black bar). MTT-reduction activities of clones #4 and #11 were negligible after irradiation relative to the dark controls. Their loss of viability thus appeared far more significant than expected from the URO levels of these two clones, as shown (Fig. 2A–C, Clones #4 and #11). This incongruity was taken to indicate that cells with URO, but too little for flow cytometric detection remained susceptible to UV-inactivation. Indeed, apparently aporphyric cells were previously observed to lose flagellar motility under the illumination of fluorescent microscopy for porphyrin^19,21^. As expected, the parental WT *L. braziliensis* were true aporphyric cells, as they remained equally viable with and without UV-exposure, yielding comparable MTT reduction activities (Fig. 2D, WT *Lb* white bar vs. black bar). These results show that all doubly transfected *L. braziliensis* clones are susceptible to ALA-induced porphyrinogenesis and photo-inactivation to various extents. Clone #4 was chosen for subsequent experiments, considering that these cells were most susceptible to ALA-induced porphyrinogenesis.

### Internalization of UV-inactivated porphyric *L. braziliensis* (Lb) by mouse bone marrow-derived macrophages (BMDM) in vitro

**Figure 3:**
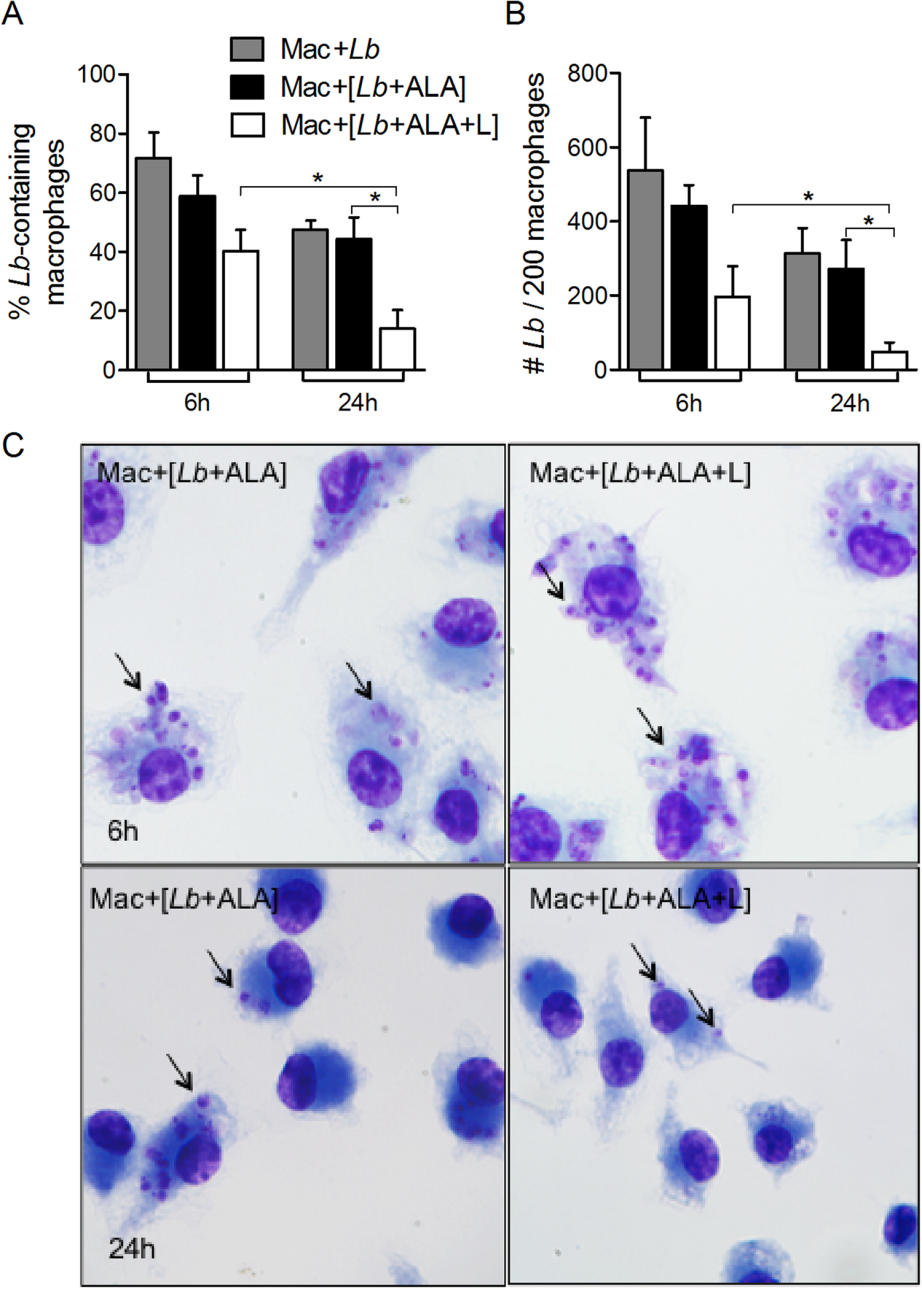
Uptake of photo-inactivated porphyric *L. braziliensis* by primary macrophages. Bone marrow-derived macrophages (BMDM) were co-cultured with *L. braziliensis* (*Lb*) transfectants (**grey bar**), those rendered porphyric by ALA-exposure (**black bar**) and those photo-inactivated by exposure of porphyric *Lb* to UV (+ L) for 30 min (**white bar**) at a host-parasite ratio of 1:10. Samples were taken at 6 h and 24 h for microscopy to assess: (**A**) the percentage of BMDM containing *L. braziliensis; *and (**B**) the number of *Leishmania* per 200 macrophages. Data are presented as mean ± S.D. from a representative experiment performed in quadruplicate, *p < 0.05. (**C**) Representative photomicrographs of (**A, B**). Arrow points to intracellular *Lb*.

Under the experimental conditions described, the BMDM took up all three forms of *Leishmania* used to different extents in the following order: parental WT (*Lb*) ≥ porphyric *Lb* (+ ALA) >  > UV-inactivated porphyric *Lb* (+ ALA + L), as determined by microscopic counting for the % infection (Fig. 3A) and the # *Lb*/200 macrophages (Fig. 3B) at two different time points. At 6 h, ~ 70% of BMDM were found to contain in each 2–3 WT *Lb* (gray bars). The values for both parameters were lower when using porphyric *Lb* (black bars) and the UV-inactivated porphyric *Lb* (white bars), especially for the #*Lb*/macrophage, i.e. a reduction to less than half of the values in comparison to the other two samples (Fig. 3A,B, 6 h). At 24 h, while the readings of both parameters decreased for all samples, they remained comparable between WT *Lb* (gray bars) and porphyric *Lb* (black bars), and were significantly lower for UV-inactivated porphyric *Lb* (white vs. gray bars) (Fig. 3A,B, 24 h). The differences between porphyric *Lb* and UV-inactivated porphyric *Lb* are illustrated in the representative photomicrographs (Fig. 3C). Exposure of wildtype *L. braziliensis* to UV did not affect their viability (Supplemental Fig. 1), indicating that it did not contribute directly to the reduced uptake of porphyric and UV-inactivated porphyric *L. braziliensis* by BMDM. The difference in the levels of uptake between the two is proportional to the levels of their light exposure, i.e. incidental stray light in the former case and UV illumination in the latter. Also, the quantitative data presented represent a net sum of both uptake and intracellular degradation in the acidic environment of phagosome-lysosome vacuoles. UV-inactivated porphyric cells were non-viable and thus more rapidly degraded than substantially viable porphyric cells exposed only to incipient light.

### Fate of *L. braziliensis* transfectants in BMDM with the intervention of double photo-inactivation by two different loading strategies

**Figure 4:**
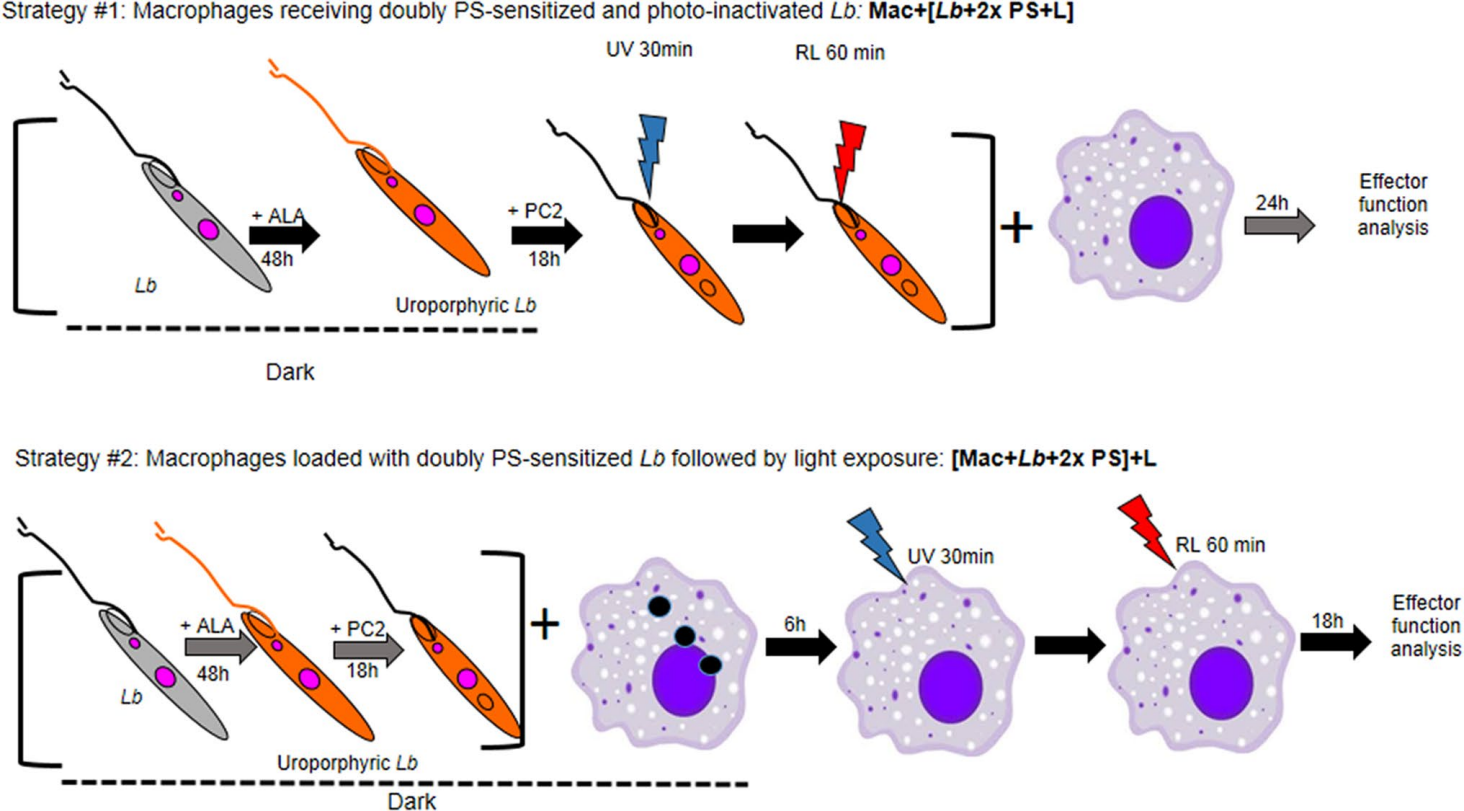
Two strategies for loading macrophages with doubly sensitized and photo-inactivated *L. braziliensis*. *Lb, L. braziliensis* transfected to express PBGD and ALAD; ALA, delta-aminolevulinate for 1st PS-sensitization by inducing cytosolic accumulation of URO; PC2, diamino-phthalocyanine for 2nd PS-sensitization via endosomal uptake; UV, Longwave UV (λmax = 366 nm) to excite URO in uroporphyric *Lb*; and RL, red light (λmax =  ~ 600 nm) to excite PC2 in the endosomes of *Lb*.

**Figure 5:**
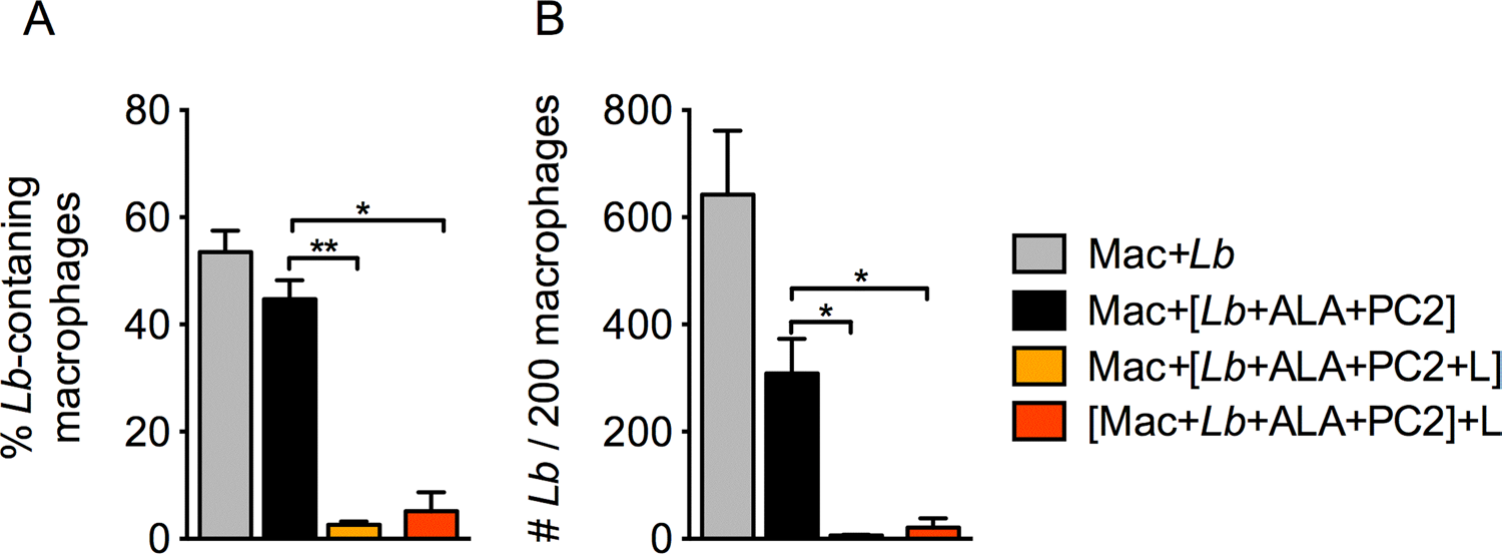
Uptake of doubly PS-sensitized and photo-inactivated *L. braziliensis* by primary macrophages. Bone marrow-derived macrophages (BMDM) were exposed at a host-parasite ratio of 1:10 to **URO/PC2** doubly sensitized parasites with (**orange bar, Mac + [Lb + ALA + Pc2 + L**]) and without (**black bar, Mac + [Lb + ALA + PC2**]) prior exposure to UV + red light. In addition, transfectant parasites were doubly sensitized and delivered to macrophages, which were then exposed to UV/red light after infection (**red bar, [Mac + Lb + ALA + Pc2] + L**). Macrophages infected with untreated *Lb* served as controls (**grey bar, Mac + Lb**). Cell samples were examined after incubation for 24 h by microscopy to assess: (**A**) the percentage of *Lb*-containing macrophages and (**B**) the number of *Leishmania* per 200 macrophages. Data are presented as mean ± . S.D. from a representative experiment performed in quadruplicate, *p < 0.05; **p < 0.01.

We first established that *L. braziliensis* was susceptible to loading with PC2 for inactivation by red light, as found with other *Leishmania* species^22,23^. By MTT reduction assays, both PC2-loaded WT *L. braziliensis* and porphyrinogenic transfectants were found to lose their viability in a concentration dependent manner after red-light exposure in contrast to those kept in the dark (Supplemental Fig. 2, white vs. black bars). PC2 was thus used to sensitize uroporphyric clone #4 for double photo-inactivation to examine their interactions with BMDM according to the two loading strategies presented in Fig. 4. Both strategies inactivated *Lb* effectively, rendering them susceptible to degradative pathways of BMDM, as indicated by a significant reduction of intracellular parasites when assessed ~ 1 day after loading (Fig. 5). Doubly PS-sensitized, but not light-inactivated clone #4 and wildtype gave a comparable rate of infection at ~ 50% (Fig. 5A gray bar vs black bar), but the average number of parasites per cell was twice fewer in the former than in the latter, i.e. 1.5 and 3 after 24 h (Fig. 5B gray bar vs black bar). While the doubly sensitized clone #4 *Leishmania* were not specifically illuminated, they were unavoidably exposed to incipient dimmed room light, accounting for the decreased values shown. The values for both parameters were significantly lowered upon loading BMDM with photodynamically inactivated parasites via both strategies (Fig. 5A,B, orange and red bars).

### Innate responses of BMDM enhanced by their interactions with doubly photo-inactivated Leishmania

**Figure 6:**
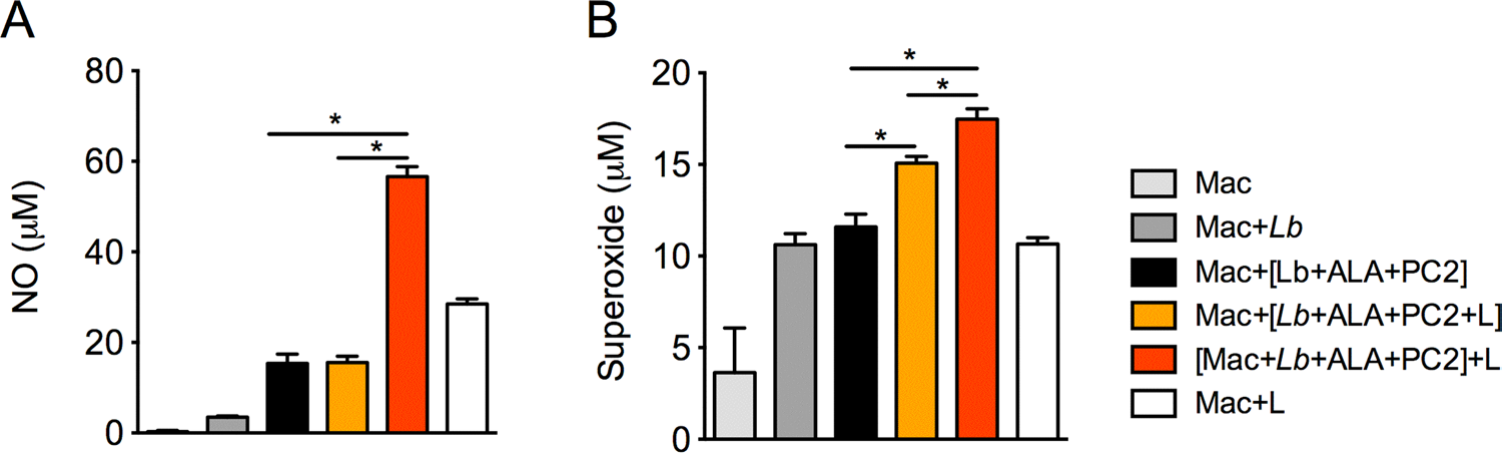
Respiratory burst of macrophages after loading with doubly PS-sensitized and photo-inactivated *L. braziliensis*. Bone marrow-derived macrophages (BMDM) were loaded at a host-parasite ratio of 1:10 with URO/PC2 doubly sensitized parasites with (**orange bar**, **Mac + [Lb + ALA + Pc2 + L])** and without (**black bar, Mac + [Lb + ALA + PC2])** prior exposure to UV + red light. In addition, parasites were doubly PS-sensitized and delivered to macrophages, which were then exposed to UV + red light (**red bar, [Mac + Lb + ALA + Pc2] + L**). Macrophages alone (**light grey bar, Mac**), macrophages infected with untreated *Lb* (**dark grey bar, Mac + Lb**) or exposed to light only (**white bar**, **Mac + L**) served as controls. (**A**) Nitric oxide and (**B**) superoxide levels were quantified in culture supernatants 24 h after priming of BMDM with LPS + IFN-γ. Data are presented as mean ± . S.D. from a representative experiment performed in quadruplicate, *p < 0.05.

The levels of NO varied significantly in the supernatants of BMDM under different conditions used (Fig. 6A), ranging from undetectable in untreated BMDM (light gray bar) to marginal-to-modest elevation on their exposure to WT *Lb* (dark gray bar), doubly PS-sensitized (black bar), photo-inactivated (orange bar) (Strategy #1) and light-exposure alone (white bar). Loading of BMDM by Strategy #2 (red bar) produced the highest elevation of NO, ~ threefold over all the other samples. Analysis of superoxide production under different conditions used gave generally similar outcome with variations in details (Fig. 6B). BMDM appeared to raise the production of superoxide slightly in response to different signals of perturbation, as noted by a doubling of its background level (light gray bar) by infection with the WT (dark gray bar) or PS-sensitized *Lb* (black bar), as with light treatment alone (white bar). Light exposure thus has no impact on *Leishmania* infection either directly on the viability of the promastigotes (Supplemental Fig. 1) or indirectly via its effect on macrophages (Supplemental Fig. 3), as documented previously^17,23^. A significant increase in superoxide above the levels of all these controls was observed by loading BMDM with photo-inactivated *Lb* according to both Strategies #1 (orange bar) and #2 (red bar). Taken together, the results indicate that light-inactivation of doubly PS-sensitized *Lb* already in BMDM via Strategy #2 elevates NO level, and that loading of BMDM with photo-inactivated *Lb* by either strategy induces the production of superoxide.

**Figure 7:**
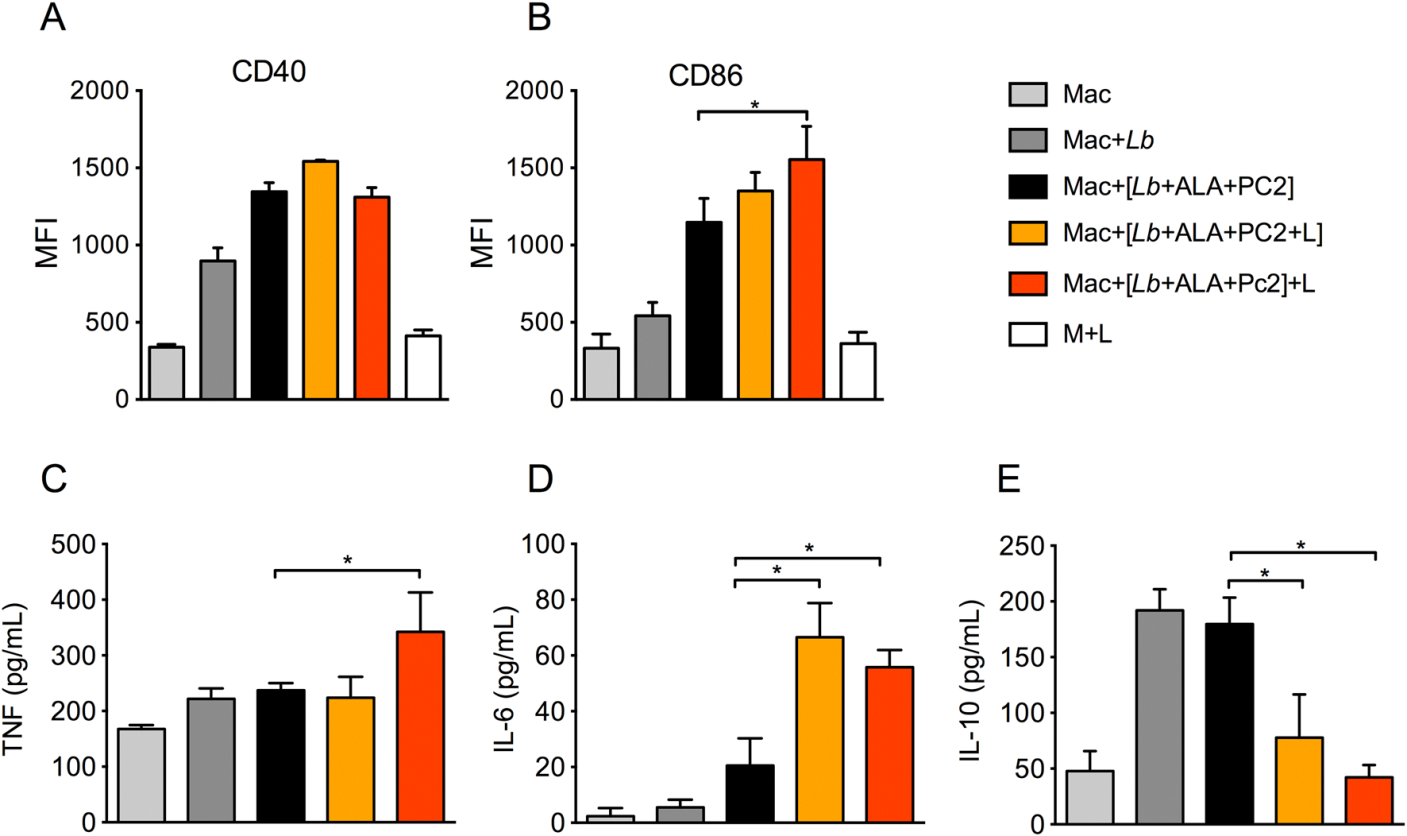
Expression of co-stimulatory molecules and cytokine production in primary macrophages after loading with doubly PS-sensitized and photo-inactivated *L. braziliensis*. Bone marrow-derived macrophages (BMDM) were exposed at a host-parasite ratio of 1:10 to URO/PC2 doubly sensitized parasites with (**orange bar**, **Mac + [Lb + ALA + Pc2 + L**]) and without (**black bar, Mac + [Lb + ALA + PC2])** prior exposure to red + UV light. In addition, parasites were doubly PS-sensitized and used to infect macrophages followed by their exposure to UV/red light after infection (**red bar, [Mac + Lb + ALA + Pc2] + L**). Macrophages alone (**light grey bar, Mac**), macrophages infected with untreated *Lb* (**dark grey bar, Mac + Lb**) or exposed to light only (**white bar**, **Mac + L**) served as controls. MFI of CD11b^+^F4/80^+^ macrophage populations were shown to express: (**A**) CD40 and (**B**) CD86, as determined by flow cytometry after incubation for 24 h. The presence of TNF (**C**), IL-6 (**D**) and IL-10 (**E**) in culture supernatants was determined by ELISA. All data are presented as mean ± . S.D. from a representative experiment performed in quadruplicate, *p < 0.05.

Loading of BMDM with photodynamically inactivated *Lb* altered the expression of co-stimulatory molecules CD40 and CD86 to different extent (Fig. 7A,B). Infection of BMDM with live PS-sensitized (black bar) and WT *Lb* (dark gray bar) elevated the MFI of both CD40 and CD86 molecules above the ground levels of BMDM alone (light gray) and those light-exposed (blank) (Fig. 7A,B). Only CD86 was significantly upregulated above these samples by loading BMDM with photodynamically inactivated *Lb* via either Strategies #1 (orange bar) or #2 (red bar).

Loading of BMDM with *Lb* photo-inactivated by both Strategy #1 (orange bar) and Strategy #2 (red bar) significantly up-regulated IL-6 over their controls, i.e. no treatment (light gray) and infection with live *Lb* (dark gray and black bar), as true for TNF via Strategy #2 (Fig. 7C,D). Significantly, the reverse was observed for IL-10 (Fig. 7E), i.e. down-regulation with photo-inactivated *Lb* (orange and red bar) and up-regulation with live *Lb* (light gray and dark bar). These results show that loading of BMDM with doubly PS-sensitized and photo-inactivated *Lb* upregulates the expression of co-stimulatory molecule CD86 and inflammatory cytokines in parallel to their intracellular processing for degradation as seen microscopically.

## Discussion

In the present study, *Leishmania braziliensis* of the *Viannia* subgenus was successfully photodynamically inactivated by the dual approach of genetic and chemical engineering, which has proven effective to render members of the *Leishmania* subgenus non-viable for safe and effective use as experimental whole-cell vaccines^12,13^. All transgenic clones produced in the present work were responsive to delta-aminolevulinate (ALA) for uroporphyrinogenesis, i.e. cytosolic de novo synthesis and accumulation of uroporphyrin 1 (URO), making them sensitive to UV inactivation. This outcome of the transfection signifies the genetic defects of *L. braziliensis* in porphyrin/heme biosynthesis, exactly as seen previously in the *Leishmania* subgenus^18,19^, i.e. the absence of the 1st five enzymes from ALA-synthase to uroporphyrinogen decarboxylase in this biosynthetic pathway. The origin of these genetic deficiencies thus appears to predate the split of the two *Leishmania* subgenera, consistent with the prevailing view for the evolutionary loss of genes coding for heme biosynthetic enzymes in trypanosomatids as a whole^24^. Of significance, not accomplished previously, is the production of as many as12 independently selected clones, which are all ALA-inducible for uroporphyrinogenesis without exception. Thus, the possibility of inadequate or incomplete transfection is effectively excluded from consideration to account for the presence of apparently aporphyric cells with levels of URO undetectable by fluorescent microscopy and flow cytometry. Pleiotropy of ALA transport and/or URO efflux^19,21^ was previously proposed to explain heterogeneity of the cell population in the % URO-positive cells and their URO levels observed during uroporphyrinogenesis. Consistent with this proposal is the observation of considerable variations noted among the 12 obtained clones. While the chosen Clone #4 stands out as the most uropohyrinogenic for UV-inactivation, its subjection to an additional round of diamino-phthalocyanine (PC2)-mediated inactivation with red light^22,23^ is for prudence to render its cell population totally non-viable with certainty. Photo-excitation of both URO and PC2 is known to produce highly destructive, but extremely short-lived ^1^O_2_ to initiate rapid oxidative inactivation of *Leishmania*^21,23,25^. By virtue of their differences in intrinsic photodynamic properties and cellular location, URO and PC2 are synergistic and compensatory to each other for effective photo-inactivation of *Leishmania* spp.^20^, including *L. braziliensis* examined in the present study.

The interactions of doubly photo-inactivated *L. braziliensis* with bone marrow-derived macrophages (BMDM) by both strategies described result in immunity-favorable outcome. In Strategy #1, loading of BMDM with non-viable clone #4 cells, which were doubly photodynamically pe-inactivated before use, spares their exposure to potentially detrimental PDT and immunosuppression known to result from infection with live promastigotes^26^. The reduced loading of BMDM with such inactivated *Leishmania* seen is fully expected, as they are non-viable and thus release no secretory products previously shown to facilitate infection of macrophages, e.g. nucleoside diphosphate kinase^27^. Strategy #2 is a trade-off between effective loadings of BMDM with URO/PC2-sensitized, but still viable *Leishmania* versus the risk of PDT collateral consequences from their exposure to light targeting *Leishmania* therein for inactivation. Irrespective of the loading strategies used, the inactivated Clone #4 cells disintegrate rapidly versus live parasites intracellularly, as indicated by comparing the loss of their structural integrity with time in BMDM. Both strategies thus produce a similarly favorable outcome in the context of loading antigen-presenting cells (APC) with whole-cell vaccines. While processing of these “vaccines” for antigen presentation to activate T cells awaits further investigation, such outcome is extrapolatable from previous findings with ovalbumin delivered to dendritic cells by photodynamically inactivated *Leishmania*^23^.

The response of BMDM to loading with doubly photodynamically inactivated *L. braziliensis* via both strategies is, for the most part, in favor of immunity (summarized in Supplemental Fig. 4). Overall, the results are consistent with and in support of the previous findings of microarray analysis in that J774 macrophages functionally returned to normalcy when their intracellular *Leishmania* were eliminated by single photodynamic inactivation via Strategy #2^17^. Here, degradation of the parasite loads in BMDM is accompanied by maintenance of effector functions, in contrast to immunosuppression known to occur in infection with live *Leishmania*^26^. Most evident for this is the reversal of cytokine profiles, i.e. the reduction of IL-10 and concomitant elevation of IL6 and TNF, the latter being pronounced in Strategy #2. These alterations are consistent with enhanced processing of antigens by the macrophages for presentation, since IL-10 is associated with non-degradative pathway favoring the survival of *Leishmania* in infected macrophages and, since TNF together with IFN-γ is known to activate macrophages with increased degradative microbicidal activities^28^. Also evident from both Strategies for photodynamic inactivation of *L. braziliensis* in BMDM is the recovery of their respiratory burst, which is countered by intricate mechanisms reported for live *Leishmania* , e.g. release of arginase to reduce nitric oxide (NO) production by substrate deprivation, up-regulation of superoxide dismutase (SOD) to reduce superoxide levels^29^ and unknown factor(s) to reduce the production of ROS by inhibiting membrane NADPH oxidase. Increased production of NO and superoxide is noted to vary with the two Strategies for photodynamic inactivation of *Leishmania* in BMDM. The elevation of NO over the control is most impressive via Strategy #2, but not Strategy #1, accountable perhaps by the difference in their loading efficiency. Superoxide is elevated similarly under both strategies as well-above the un-infected, but also, to a lesser extent, over the other control groups, i.e. live parasite infection and light exposure alone. The elevation of superoxide in BMDM to some extent in these heterogeneous control groups suggests that the production of this ROS may be subjected to the regulations by several different signal pathways. Redundant anti-oxidant systems also exist in both macrophages and *Leishmania*, presenting multi-layered complexity of the host-parasite interactions in the disposal of ROS, like superoxide. This is in contrast to ^1^O_2_, which neither macrophages nor *Leishmania* is known to produce and thus has evolved no specific mechanism for its detoxification. The examination of co-stimulating molecules is on account of *Leishmania* strategies, which have been reported to manipulate the co-stimulatory pathways of macrophages to their advantages^30–32^. Loading of macrophages with photodynamically inactivated *L. braziliensis* by both Strategies offers some evidence for upregulation of CD86 and, to a lesser extent, CD40, even though infection of BMDM with live parasites also elevate them over un-infected and light-alone controls . Up-regulation of these co-stimulatory molecules has the potential to facilitate activation of T cells via binding to their CD28 and CD40L, respectively, as previously demonstrated for dendritic cells in self-resolving *L. braziliensis* infection^33^.

The results obtained shed additional lights on the relative merits of the two strategies for vaccination, both using whole *Leishmaina* cells to retain their entire antigenic repertoire for presentation to the immune system. Strategy #1 is favorably disposed with significant advantages of preparatory simplicity relatively and safety assurance by using pre-inactivated non-viable *Leishmania.* These are the overriding issues for consideration of any vaccine development. Doubly photodynamic inactivation of *Leishmania* indeed renders them non-viable^20^, but preserves their antigenicity and adjuvanicity. The efficacy of Strategy #1 was thus demonstrated in experimental vaccination against cutaneous leishmaniasis^13^. As suggested by the in vitro work presented here with *L. braziliensis*, Strategy #1 may be further improved to enhance its efficacy by increasing doses for loading APC to match that of Strategy #2. While the latter was previously shown as effective in prophylactic vaccination against experimental visceral leishmaniasis^12^, it is burdened by significant disadvantages, i.e. the cumbersome in situ light illumination after immunization with PS-sensitized, but live *Leishmania* and the potential risk of infection due to possibility of their incomplete inactivation. In either case, photodynamically inactivated *Leishmaina* are not lesion-derived live parasites used for leishmanization^34^ and are different from other whole-cell live preparations, such as *Leishmania* attenuated spontaneously or crippled by other chemical, physical or genetic means^8–11^. Clearly, the use of doubly PS-sensitized and photo-inactivated *L. braziliensis* for immunization ameliorates the risk of their potential persistence, reversion to virulence and disease reactivation associated with use of attenuated live mutant parasites, thereby providing novel prophylaxis alternatives against leishmaniasis caused by this species.

## Methods

### Ethics statements

Female BALB/c mice, 6–8 weeks of age, were obtained from the IGM-FIOCRUZ animal facility where they were maintained under pathogen-free conditions. All animal experimentation was conducted in accordance with the Guidelines for Animal Experimentation established by the Brazilian Council for Animal Experimentation Control (CONCEA). All procedures involving animals were approved by the local Institutional Review Board for Animal Care and Experimentation (CEUA-IGM-FIOCRUZ-003/2014.

### Cells

*Leishmania braziliensis* promastigotes (MHOM/BR/00/BA788)^35^ were maintained in Schneider’s medium (SIGMA) supplemented with 10% heat-inactivated FCS, 100 U/ml penicillin, 100 μg/ml streptomycin (all from Invitrogen) or in Medium 199 (SIGMA) supplemented with 20% heat-inactivated fetal calf serum (FCS), Hepes (40 mM, pH 7.4), Adenine (0.1 mM), Hemin (5 μg/ml), Biotin (1 μg/ml) and antibiotics (penicillin 100 IU/ml and streptomycin 100 µg/ml) (all from Invitrogen). Uroporphyrinogenic *L. amazonensis* were grown under similar conditions with 20 ug/ml tunicamycin and 100 ug/ml G418^19^.

Primary macrophages differentiated from mouse bone marrow as previously described^36^ were maintained in RPMI 1640 medium (SIGMA) supplemented with 100 U/ml penicillin, 100 ug/ml streptomycin and 10% FCS.

### Construction of expression vectors

Two different plasmids were used for double transfection of *L. braziliensis* promastigotes to express mammalian cDNA encoding ALAD and PBGD^19^, i.e. pX-NEO-*alad* available previously (Supplemental Fig. 5A) and the newly constructed pXG-HYG-*pbgd*. For the latter, p6.5-*pbgd*^19^ was first *BamHI*-digested and the *pbgd* coding sequence released was gel-purified for cloning at the *BamHI* sites of the pXG-HYG expression vector (Supplemental Fig. 5B). Competent *E. coli* (DH5α) cells were transformed with the constructs and plasmids from several colonies were restriction-mapped to select for clones with correctly oriented insert.

### Transfection of *L. braziliensis* and selection of transfectants

Promastigotes of *L. braziliensis* were subjected to two consecutive rounds of electroporation to obtain double transfectants expressing both ALAD and PBGD. The first round used pX-NEO-*alad* and the resulting single transfectants were subjected to a second round of electroporation with the newly constructed pXG-HYG-*pbgd,* thereby producing *L. braziliensis* double transfectants expressing both ALAD and PBGD.

For electroporation, promastigotes grown to mid-log phase were harvested by centrifugation for resuspension to a cell density of 10^8^ cells/ml in Tb-BSF electroporation buffer^37^. After electroporation, transfectants were selected on 96-well tissue culture plates using the limiting dilution method in M199 medium supplemented with appropriate antibiotics. For the first round of transfection, mid-log cell suspension (0.4 ml) was mixed with pX-NEO-*alad* (5 μg) in a pre-chilled 2 mm electroporation cuvette (SIGMA) for electroporation using a Bio-Rad Gene Pulser^38^. A clone well adapted to G418 selection was expanded for a second round of transfection with pXG-HYG- *pbgd* by electroporation, as described above. Doubly transfected cells were selected for resistance to both hygromycin (HYG) and G418 and cloned in 96-well tissue culture plates with culture medium supplemented with G418 and HYG at 50 μg/ml each. The cultures were monitored for 2–4 weeks. Transfectants emerging in wells containing cells at the highest dilutions were further expanded in selective medium with increasing drug pressures, i.e. G418 and HYG at 100 ug/ml each. Double transfectant clones grew stably in Schneider’s medium under the selective pressure of both drugs at the concentration, as indicated.

### Aminolevulinate-induced porphyrinogenesis of genetically complemented *L. braziliensis* clones

Transfectants from 12 independent clones were each grown to stationary phase. These cells were harvested, washed and resuspended to 5 × 10^7^ cells/ml in RPMI 1640 (Invitrogen)/0.01% bovine serum albumin (RPMI-BSA). The cells were exposed at 26 °C for up to 48 h in the dark to 1 mM ALA (SIGMA). ALA-exposed and control cells were washed and examined by phase contrast for their integrity. Porphyrin fluorescence of these cells was assessed in three different ways. Aliquots of cell suspension were sedimented for illumination in the dark with an UV-lamp (λmax = 366 nm) to check for porphyrin fluorescence in the cell pellets and in the supernatants for porphyrin release. Microscopically, live cell samples were examined for porphyrin by excitation with Krypton/argon-mixed gas laser at 488 nm and filtered for emission at 605 nm in an Olympus FluoView confocal microscope. ALA-exposed cells of all 12 clones and controls were sampled at different times to assess the kinetics of their porphyrinogenesis by flow cytometry (BD-LSR Fortessa) at an excitation wavelength of 405 nm and emission wavelength of 610 nm. Data from 30,000 events were acquired to determine the % of porphyrin-positive population for each clone. Data were analyzed using FlowJo (Version 10.2). Parental wild-type *L. braziliensis* and *L. amazonensis* porphyrinogenic mutants^19^ exposed to ALA were included as negative control and positive control, respectively.

### Photo-inactivation of porphyric *L. braziliensis* transfectants and MTT reduction assay to assess their viability

After ALA treatment in the dark, the 12 clones of double transfectants were each suspended to 5 × 10^7^ cells/ml in RPMI-BSA and placed at 500 µl/well in 24-well culture plates. Two sets were prepared for each clone. One set was exposed with lid off to longwave UV (λmax = 366 nm) from the top for 30 min. The second set was kept under identical conditions, but foil-wrapped to keep cells in the dark. All samples were subsequently incubated overnight and assessed for cell viability by MTT (3-(4,5-dimethylthiazol-2-yl)-2,5-diphenyltetrazolium bromide) reduction assay (SIGMA) according to the manufacturer’s protocol. Parental wild-type cells of *L. braziliensis* were included as additional controls.

### In vitro uptake of photo-inactivated porphyric *L. braziliensis* and controls by bone marrow-derived macrophages (BMDM)

The primary macrophages obtained from mouse bone marrow were suspended in culture medium for seeding at 3 × 10^5^ cells/500 μl/coverslip per well in 24-well plates. Monolayers of adherent macrophages formed on the coverslips were washed to remove non-adherent cells and exposed to experimental or control *L. braziliensis* promastigotes at 10:1 parasite/host cell ratio (clone #4), i.e. 3 × 10^6^/500 μl in RPMI 1640 + 10% FBS/well. The plate cultures were incubated at 35 °C, 5% CO_2_. After incubation for 6 and 24 h, coverslip samples were withdrawn in quintuplicate, each extensively washed to remove non-internalized *Leishmania*, methanol-fixed and stained with hematoxylin and eosin. *Leishmania* loading or infection of macrophages was assessed by scanning 200 macrophages in each sample for enumeration of cells with and without *Leishmania*, and the total number of intracellular parasites by microscopy.

### Exogenous sensitization of *L. braziliensis* with diamino-phthalocyanine (PC2) for their photo-inactivation with red light

Porphyrinogenic and porphyric *L. braziliensis* (clone #4) were sensitized with PC2 as previously described^22^. Promastigotes were grown to late-log phase, washed and resuspended at 5 × 10^7^ cells/ml in RPMI-BSA, in presence of PC2 at graded concentrations of 0.001 to 5 μM. Cells were exposed to 0.5% DMSO, equivalent to that of the highest PC2 concentration used, to serve as the solvent control. After overnight incubation at 26 °C in the dark, PC2-sensitized and un-sensitized control cells were washed and resuspended to its original cell density for exposure to red light (RL) from the bottom of the culture plates for 60 min until promastigotes ceased flagellar motility (1–2 J/cm^2^)^19,21,23^. All cell samples were assessed for their viability by MTT reduction assay, as described in the preceding section.

### Double PS-sensitization and photo-inactivation of *L. braziliensis*

Porphyrinogenic *L. braziliensis* promastigotes (clone #4) were subjected to double PS-sensitization and photo-inactivation at 5 × 10^7^/ml in RPMI-BSA. These cells were sensitized first cytosolically with porphyrin by ALA exposure and then endosomally with PC2 (1 µM), as described above. These doubly sensitized cells were washed and resuspended in RPMI-BSA for photo-inactivation under above-described conditions, i. e. exposure first with lid off to longwave UV (λmax = 366 nm) from the top for 30 min and then to red light (λmax =  ~ 600 nm) from the bottom for 60 min (1–2 J/cm^2^).

### Effector responses of bone marrow-derived macrophages (BMDM) to doubly PS-sensitized and photo-inactivated *L. braziliensis*

BMDM were seeded in 24-well culture plates at a density of 10^6^ cells per well. Cells were then incubated with porphyrin-PC2 doubly sensitized *L. braziliensis* using two different strategies for photo-inactivation (Fig. 4). In Strategy #1, macrophages were loaded with doubly-sensitized and pre-photo-inactivated *L. braziliensis* for 6 and 24 h (**Mac + [Lb + 2 × PS + L]**). In Strategy #2, macrophages were first infected with the doubly PS-sensitized *L. braziliensis* for 6 h and then exposed post-infection to longwave UV and red light. After illumination, cells were cultured for another 18 h (**[Mac + Lb + 2 × PS] + L**).

### Quantitative analysis of nitric oxide, superoxide and cytokines in culture supernatants of BMDM loaded with photo-inactivated transfectants

BMDM were primed for 24 h with LPS (40 ng/mL) and IFN-γ (10 ng/mL) (all from Invitrogen) or with 0.5 mM hydroxylamine hydrochloride (Acros Organics) for determining NO and superoxide, respectively. BMDM were then loaded with transfectants according to strategies #1 and #2 (Fig. 4). Cell culture supernatants were collected after 18 h for NO and superoxide quantification using Griess reagent (**20**). Cytokine levels in the culture supernatants collected were determined by ELISA, following manufacturer’s instructions (eBioscience).

### Flow cytometric analysis of BMDM for co-stimulating molecules after exposure to doubly PS-sensitized and photo-inactivated *L. braziliensis*

BMDM were exposed to *L. braziliensis* according to strategies #1 and #2 (Fig. 4) for 18 h in 5 ml polypropylene tubes. Cell samples were harvested for staining with fluorochrome-labeled antibodies specific to F4/80, CD11b, CD40 and CD86 (Filters used: PE, PE/Cy7, FITC and BV 421, respectively) according to established protocols. Briefly, cells were first blocked at 4 °C with rat anti-mouse CD16/32 (5 mg/ml; BD Pharmingen) for 10 min and then stained with anti-mouse antibodies for 30 min (1:200 dilution each molecule) at 4 °C. Cells were also stained with Fixable Viability Dye (eBioscience/ThermoFisher) to recognize dead cells for exclusion. Cells were finally fixed with 2% paraformaldehyde in PBS for 10 min, washed by centrifugation with washing buffer and kept at 4 °C in the dark until data acquisition. Fluorescence-minus-one (FMO) controls for CD40 and CD86 were included in all experiments. Data were acquired in a Fortessa flow cytometer (BD Biosciences, USA) and analyzed by using FlowJo software (Tree Star, Version 10.2). Macrophages were pre-gated by surface phenotype (F4/80 and CD11b positive cells).

### Statistical analysis

Comparisons between two groups were performed by using Mann–Whitney (non-parametric *t* test) and among more than two groups by using Kruskal Wallis tests. Analyses were conducted using Prism (GraphPad, V 5.0) and a *p* value ≤ 0.05 was considered significant. Data are presented as mean ± standard deviation.

## Supplementary information

Supplementary Information.
