# Supplementary material for: Photodynamic inactivation of Leishmania braziliensis doubly sensitized with uroporphyrin and diamino-phthalocyanine activates effector functions of macrophages in vitro
Source: Sci Rep. 2020 Oct 13;10:17065. doi: 10.1038/s41598-020-74154-1 (PMC7555832; doi:10.1038/s41598-020-74154-1)
Supplement: Supplementary file 1 — Supplementary Information. [file 41598_2020_74154_MOESM1_ESM.docx]

**Supplementals for:**

**Photodynamic inactivation of *Leishmania braziliensis* doubly sensitized with uroporphyrin and diamino-phthalocyanine activates effector functions of macrophages *in vitro***

Rohit Sharma^1^, Sayonara M. Viana^1^, Dennis K. P. Ng^2^, Bala K. Kolli^3^, Kwang Poo Chang^3^, Camila I. de Oliveira^1,4^*

**Supplemental Figure 1**: **Insensitivity of *Leishmania brazilensis* to longwave UV exposure under the conditions used.**

Promastigotes of *L. braziliensis* (**Lb**) were grown to stationary phase, harvested and resuspended in PBS. Aliqouts of the cell suspensions with or withhout exposure to longwave UV for 30 min were incubated overnight and sbjected to MTT reduction assay under the conditions as described in Materials and Methods. Experiments were performed in quadruplicate and data presented as mean OD unit ± S.D.


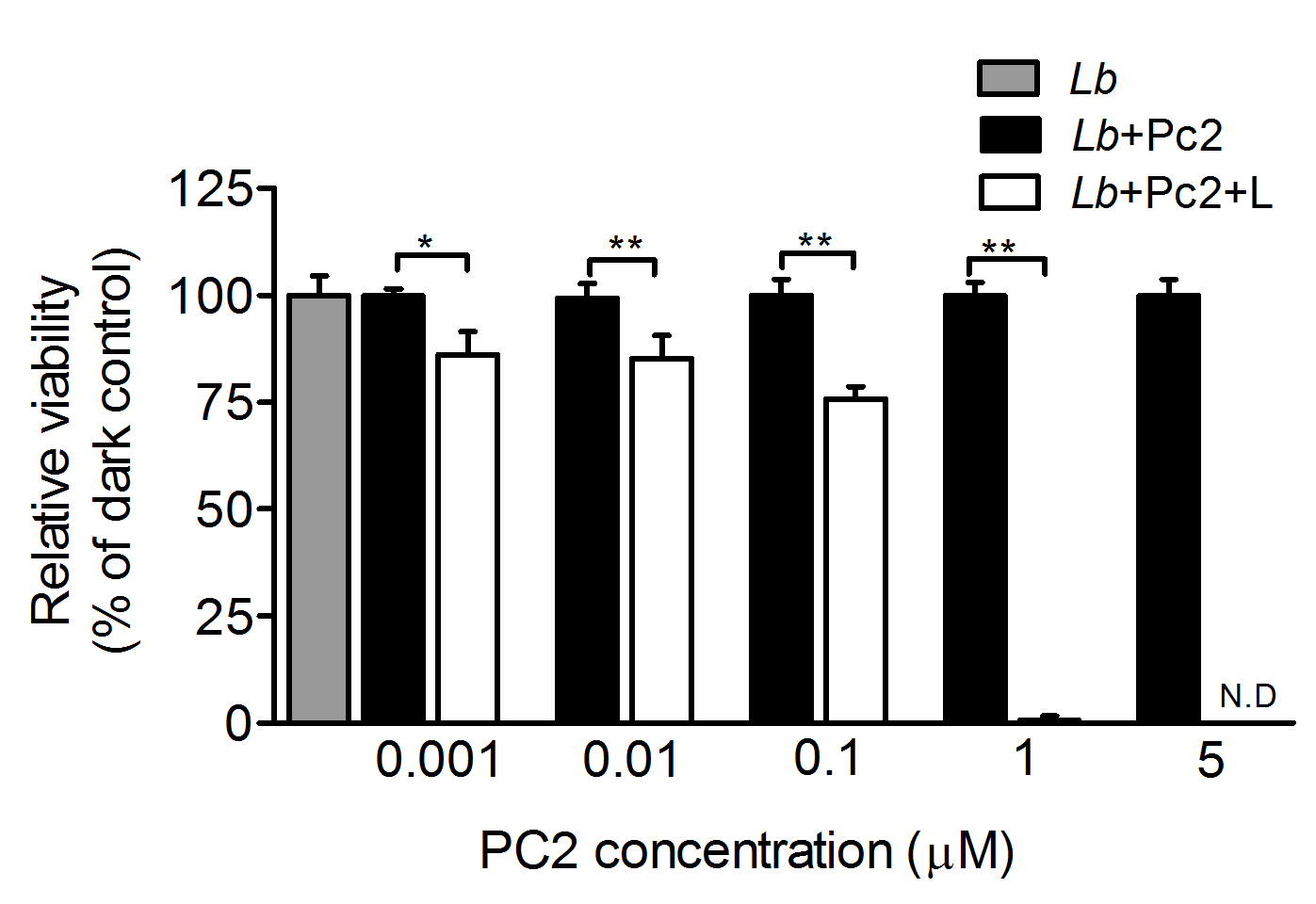


**Supplemental Figure 2. PC2-mediated photo-inactivation of *L. braziliensis*.**

Promastigotes (*Lb*) were incubated in the dark with PC2 at various concentrations as indicated for 18h (**dark bar, *Lb*+PC2**) and exposed to red-light (L) for 60 min. WT *L. braziliensis* cells (**grey bar, *Lb***), those sensitized with graded concentrations of PC2 in the dark (**black** **bar**) and the latter group after light exposure (**white bar, *Lb*+PC2+L**) were assessed for viability by their MTT reduction activities, the values being expressed in % of dark controls. Data are presented as mean ± S.D. from a representative experiment performed in quadruplicate. ** *p*<0.01; **p*<0.05. ND = Not detectable.

**Supplemental Figure 3:** **Insensitivity of bone marrow-derived macrophages to light exposure under the conditions used.**

Bone marrow derived macrophages (**Mac**) with (**blank bar**) and without (gray bar) exposure to longwave UV for 30 min and red light for 1h (**+L**) were incubated overnight and subjected to MTT reduction assay under the conditions as described in the Materials and Methods. Experiments were performed in quadruplicate and data presented as mean OD unit ± S.D.


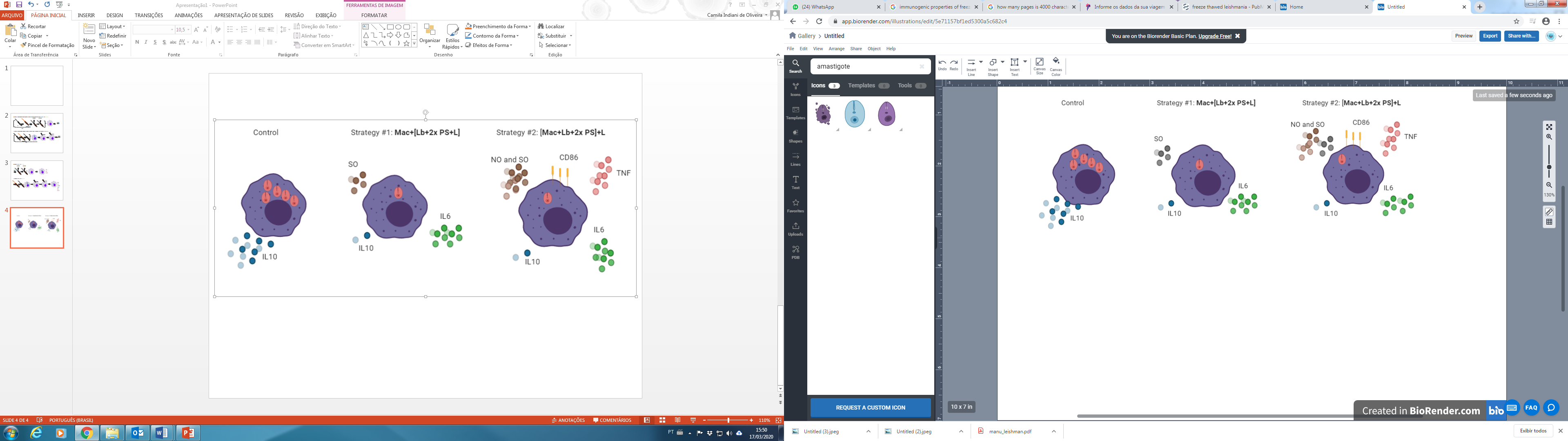
 **Control Strategy #1: Mac+[Lb-2XPS+L] Strategy #2: [Mac+Lb+2XPS]+L**

**Supplemental Figure 4**: **Up-regulation of macrophage effector functions by loading with *Leishmania braziliensis* photodynamically inactivated by two different strategies.**

Infection of bone marrow-derived macrophages (**Mac**) with live *L. braziliensis* (**Lb**) increases their release of IL10 (**Control**). Loading of Mac with photodynamically inactivated Lb up-regulates the production of superoxide (**SO**) and IL-6, and down-regulates that of IL-10 (**Strategy#1**). Infection of Mac with 2Xphoto-sensitized Lb followed by their light-inactivation *in situ* also dowon-regulates IL10, but up-regulate nitric oxide (**NO**) , TNF and CD86 in addition to IL-6 (**Strategy #2**). Intracellular Lb, Orange; Extracellular mediators, circles in different colors and shades.

A

M


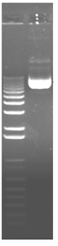


M


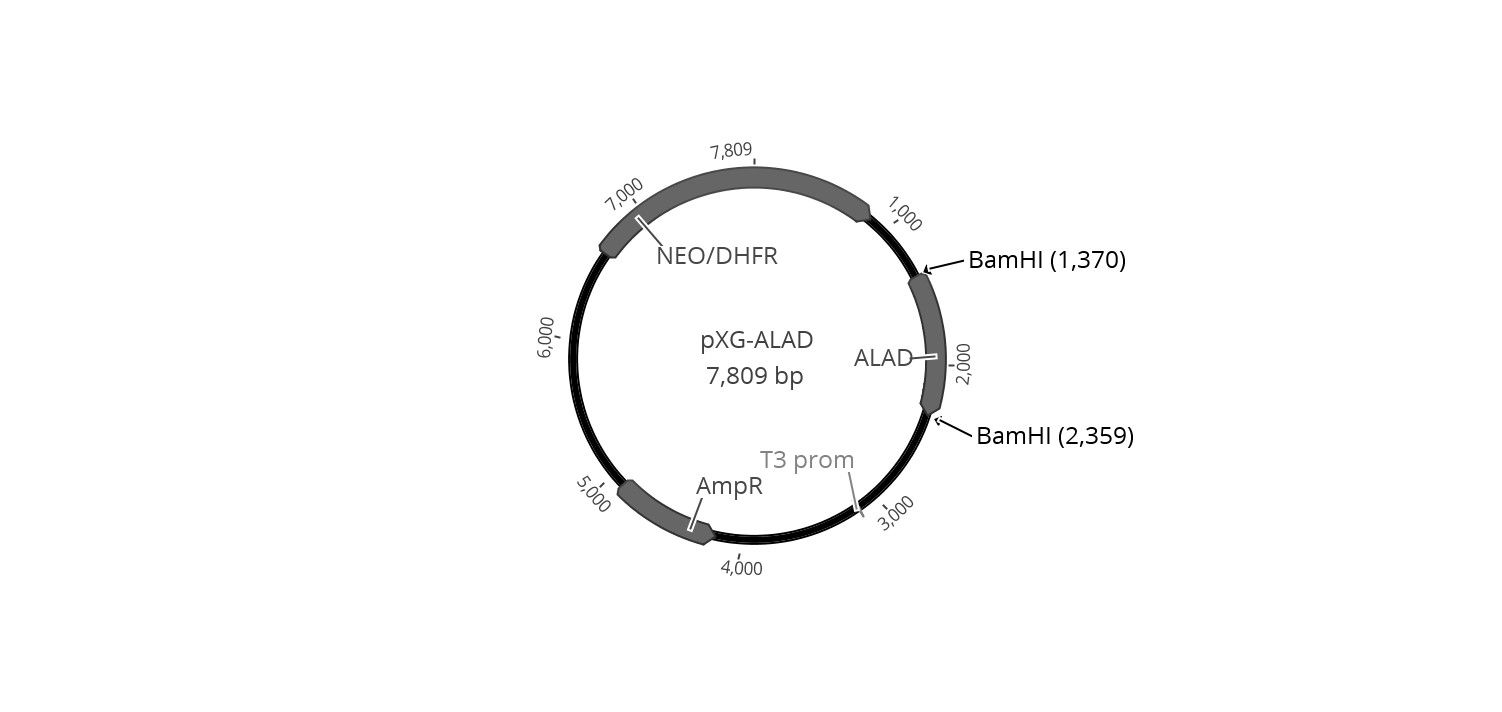


B


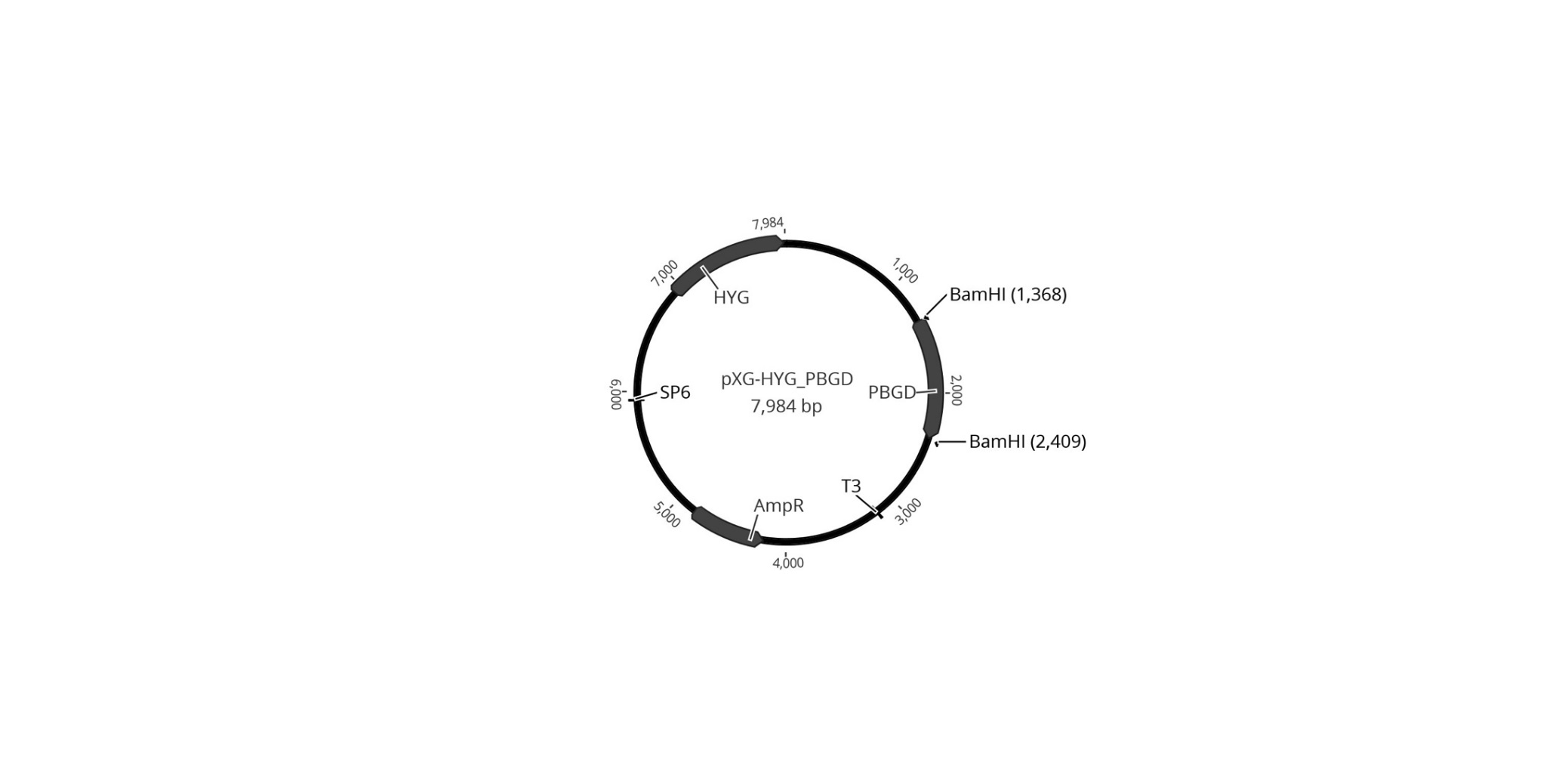

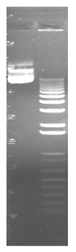


M

**Supplemental Figure 5. Plasmid maps of PBGD and ALAD expression constructs used for genetic complementation of *L. braziliensis*.**

pX-NEO-alad **[A]** and pXG-HGY-pbgd **[B]**, ~ 8 kb each, were constructed for sequential transfection of promastigotes to express ALAD and PBDG. Both plasmids migrated in 0.8% agarose gel as circular DNA expected. Lane M, 1-kb DNA marker.
